# Editorial: Endocrine and metabolic consequences of childhood obesity, volume II

**DOI:** 10.3389/fendo.2023.1239914

**Published:** 2023-07-21

**Authors:** Artur Mazur, Dénes Molnár, Aneta Monika Gawlik, Grzegorz Telega, Elpis Vlachopapadopoulou, Malgorzata Wojcik

**Affiliations:** ^1^ Department of Pediatrics, Pediatric Endocrinology and Diabetes, Medical Faculty, University of Rzeszów, Rzeszów, Poland; ^2^ Department of Pediatrics, Medical School, University of Pécs, Pécs, Hungary; ^3^ National Laboratory for Human Reproduction, Szentágothai Research Centre, University of Pécs, Pécs, Hungary; ^4^ Department of Pediatrics and Pediatric Endocrinology, Faculty of Medical Sciences, Medical University of Silesia, Katowice, Poland; ^5^ Division of Gastroenterology, Hepatology and Nutrition, Children's Hospital of Wisconsin, Medical College of Wisconsin, Milwaukee, WI, United States; ^6^ Department of Endocrinology, Panagiotis & Aglaia Kyriakou Children’s Hospital, Athens, Greece; ^7^ Department of Pediatric and Adolescent Endocrinology, Chair of Pediatrics, Pediatric Institute, Jagellonian University Medical College, Krakow, Poland

**Keywords:** children, obesity, hypertension, CVD, insulin resistance

Obesity – a pandemic of the twenty-first century – is one of the greatest public health problems worldwide. Overweight and obesity affect nearly one in five children in the world and one in three in Europe (1). Recent estimates suggest that overweight and obesity is the fourth most common risk factor for noncommunicable diseases in Europe, following hypertension, dietary risk factors and tobacco use. Moreover, obesity during adolescence increases the risk for cardiovascular disease and premature death during adulthood, independently of the persistence of obesity in adulthood (2, 3). However, improvement of weight control and normalization of BMI in adolescence diminishes the risk of developing DM2 in adulthood (3). Certain comorbidities such as type 2 diabetes mellitus (T2DM) and metabolic associated fatty liver disease, until recently considered “adult diseases”, are now frequently encountered in children with obesity (2, 4). Evaluation and monitoring for these comorbidities are important components of health care for children the growing impact of childhood obesity on the development of short- and long-term endocrine and metabolic complications.

No single cause is responsible for increased incidence of childhood obesity. It cannot be blamed on genetics factors or environment factors alone. In this Research Topic our contributors explore the mechanisms behind, linking intrauterine, postnatal, and early childhood metabolic environment to obesity and its complications (2, 5, 6).

Nakhleh et al. revealed that class 1 obesity in children’s and adolescents (BMI ≥ 110% of the 95th percentile) was associated with higher prevalence and clustering of cardiometabolic risk factors.

Rajamoorthi et al. highlighted the role of the environmental factors, including the globalization of the western diet and unhealthy lifestyle choices. In an elegant review they argued that starting from conception type and timing such exposures come into play impacting on the overall risk of obesity and future adverse health outcomes.

An important new observation was reported by Seget et al. as they documented that the prevalence of obesity is increasing among in children with diabetes mellitus type 1 (T1DM) and may influence the glycemic control.

On the other hand, Pixner et al. investigated LACA and its mediators (amino acids and glucagon), focusing on the relationship between glucose and the LACA in adult and pediatric subjects.

Kącka et al. introduced novel markers of metabolic complications in obese T1DM and non-diabetic subjects.

Analysis of the taste preferences and sensitivity of mothers and their children in the relation to excessive body weight of children is presented by Sobek and Dąbrowski in article “*Lifestyle intervention changes are crucial in the prevention and treatment of childhood obesity*”.

Strączek et al. found that one-year dietary education resulted in significant improvements in body weight, waist, and hip circumference, WHtR and selected measured carbohydrate and lipid metabolism parameters with the exception of total cholesterol. The one-year dietary intervention did not have the same effect on the change in dietary habits in children and in their mothers.

The assessment of childhood obesity comorbidities and risk of its complication is challenging and difficult. de Lamas et al. concluded that controlling obesity and cardiometabolic risk factors, especially insulin resistance and blood pressure in children during the prepubertal stage appears to be effective in prevention of pubertal metabolic syndrome.

Artemniak-Wojtowicz et al. experimentally proved that weight reduction leads to significant decrease of circulating Th17 cells and improvement of lipid parameters. This significant reduction of proinflammatory Th17 cells is a promising finding suggesting that obesity-induced inflammation in children could be reversible.

One of the key problems in the development of obesity complications is the liver involvement. Liver abnormalities - collectively known as metabolic associated fatty liver disease is becoming a more prevalent clinical problem, in obese children and adolescents. Maruszczak et al. described determinants of hyperglucagonemia in Pediatric Non-Alcoholic Fatty Liver Disease. Brunnert et al. revealed usefulness of the liver stiffness measurement in the evaluation of liver involvement in obese adolescents. Furdela et al. revealed that triglyceride glucose index, pediatric NAFLD fibrosis index, and triglyceride to high-density lipoprotein cholesterol ratio are a valuable combination of predictive markers of metabolically unhealthy phenotype in Ukrainian overweight/obese boys.

Obesity can also associate with complications of calcium-phosphorus and bone metabolism regulation (5). That was investigated by Erazmus et al. in the article “*Decreased level of soluble Receptor Activator of Nuclear Factor-κβ Ligand (sRANKL) in overweight and obese children*”.

Krajewska et al. confirmed that vitamin D has positive effect on metabolic profile in overweight and obese children, but the relationship between vitamin D and chemerin is not clear.

Zembura and Matusik found that sarcopenic obesity is highly prevalent in children and adolescents and is associated with various adverse health outcomes including significant association with cardiometabolic outcomes, severity of non-alcoholic fatty liver disease (NAFLD), inflammation, and mental health. Findings of this review highlight the need for the development of a consensus regarding definition, standardized evaluation methods, and age and gender thresholds for sarcopenic obesity for different ethnicities in the pediatric population.

Many factors influencing the development of obesity and its complications are still unknown. Future studies are needed to elucidate many questions and concerns raised by our contributors. Nevertheless, we do hope that readers will find our Research Topic informative and inspiring.

## Author contributions

AM, MW - writing draft of manuscript. AG, GT, EV - review. DM - final correction and approval.
